# Evaluating the Impact of Low-Pathogenicity Avian Influenza H6N1 Outbreaks in United Kingdom and Republic of Ireland Poultry Farms during 2020

**DOI:** 10.3390/v16071147

**Published:** 2024-07-16

**Authors:** Michael J. McMenamy, Robyn McKenna, Valerie B. Bailie, Ben Cunningham, Adam Jeffers, Kelly McCullough, Catherine Forsythe, Laura Garza Cuartero, Orla Flynn, Christina Byrne, Emily Connaghan, John Moriarty, June Fanning, Stephanie Ronan, Damien Barrett, Alice Fusaro, Isabella Monne, Calogero Terregino, Joe James, Alexander M. P. Byrne, Fabian Z. X. Lean, Alejandro Núñez, Scott M. Reid, Rowena Hansen, Ian H. Brown, Ashley C. Banyard, Ken Lemon

**Affiliations:** 1 Virological Molecular Diagnostics Laboratory, Virology Branch, Agri-Food and Bioscience Institute, Stoney Road, Stormont, Belfast BT4 3SD, UK; 2 Disease Surveillance & Investigation Branch, Agri-Food and Bioscience Institute, Stoney Road, Stormont, Belfast BT4 3SD, UK; 3 Central Veterinary Research Laboratory, DAFM, Backweston Campus, Stacummy Lane, W23 X3PH Celbridge, Ireland; 4 National Disease Control Centre, Department of Agriculture, Food and the Marine, Agriculture House, Kildare Street, D02 WK12 Dublin, Ireland; 5 European Union Reference Laboratory (EURL) for Avian Influenza and Newcastle Disease, Istituto Zooprofilattico Sperimentale delle Venezie, 35020 Padua, Italy; 6 Department of Virology, Animal and Plant Health Agency—Weybridge, Woodham Lane, New Haw, Addlestone KT15 3NB, UK; 7 Pathology and Animal Sciences Department, Animal and Plant Health Agency—Weybridge, Woodham Lane, New Haw, Addlestone KT15 3NB, UK; 8 Now the Pirbright Institute, Ash Road, Woking GU24 0NF, UK

**Keywords:** low-pathogenicity avian influenza virus, avian influenza virus subtype H6, H6N1 outbreaks, impact of LPAIV

## Abstract

In January 2020, increased mortality was reported in a small broiler breeder flock in County Fermanagh, Northern Ireland. Gross pathological findings included coelomitis, oophoritis, salpingitis, visceral gout, splenomegaly, and renomegaly. Clinical presentation included inappetence, pronounced diarrhoea, and increased egg deformation. These signs, in combination with increased mortality, triggered a notifiable avian disease investigation. High pathogenicity avian influenza virus (HPAIV) was not suspected, as mortality levels and clinical signs were not consistent with HPAIV. Laboratory investigation demonstrated the causative agent to be a low-pathogenicity avian influenza virus (LPAIV), subtype H6N1, resulting in an outbreak that affected 15 premises in Northern Ireland. The H6N1 virus was also associated with infection on 13 premises in the Republic of Ireland and six in Great Britain. The close genetic relationship between the viruses in Ireland and Northern Ireland suggested a direct causal link whereas those in Great Britain were associated with exposure to a common ancestral virus. Overall, this rapidly spreading outbreak required the culling of over 2 million birds across the United Kingdom and the Republic of Ireland to stamp out the incursion. This report demonstrates the importance of investigating LPAIV outbreaks promptly, given their substantial economic impacts.

## 1. Introduction

Avian influenza viruses (AIVs) are important avian pathogens that cause significant losses within the poultry industry globally. Depending on subtype, AIVs can spread through populations, in either the presence or absence of clinical disease. Although not fully defined, the outcomes of infection are likely a factor of species susceptibility (including host, nutritional, immune status, age, and presence of intercurrent disease) and the genetics of the agent involved (pathotype, virulence factors, infectivity). These viruses circulate freely within wild bird reservoirs, mixing genetic material during coinfection events to drive the emergence of novel reassortments [1,2,3].

AIV infection of poultry is categorised as either high-pathogenicity (HP), generally resulting in high morbidity and mortality rates, or low-pathogenicity (LP), where disease outcomes can range from asymptomatic to invasive and systemic depending on the AIV strain involved [4]. The significance of outbreaks to the poultry sector is generally linked to viral factors including the genetics of the haemagglutinin (HA) gene and virulence markers within. There are 16 haemagglutinin (HA) and nine neuraminidase (NA) subtypes of AIVs that infect birds, with HA and NA viral glycoproteins driving both entry and exit from host cells [5]. The H5 and H7 HA subtypes are notifiable in poultry in the United Kingdom (UK), and control/notification of LP viruses in other countries depends on local legislation recognising they can be associated with the emergence of HPAIV. In nature, HPAIV can evolve from LP avian influenza (LPAI) progenitor strains of the H5 and H7 subtypes following circulation in poultry [6]. In addition, reassortment between HPAIVs and LPAIVs can generate novel viruses with potentially altered biological properties. Active AIV surveillance in poultry focuses on negation of the H5 and H7 subtypes. The detection of HPAIV as defined in the WOAH manual [7] in the UK results in the culling of all poultry within a premises, as defined by legislative requirements. The economic losses from culling of birds on infected premises (IPs) can be profound. Immediate culling of stock is followed by a cleansing and decontamination process before tentative site re-stocking can progress, with one option being to leave units free from birds for a 12-month period. The impact on livelihoods following an outbreak can therefore be considerable. Control of LP H5 and H7 will depend on national legislation; however, LPAIVs of other subtypes are not usually subject to official control, and where events occur is usually the industry’s responsibility to manage.

Influenza A viruses have a segmented genome, consisting of eight separate genetic segments. In addition, the RNA-dependent RNA polymerase is error-prone, lacking proofreading ability. These two factors contribute to the continued evolution and rapid emergence of novel influenza A viruses [8]. This evolutionary process can result from two key activities—genetic drift, whereby the accumulation of mutations can lead to altered species tropism or virulence, and genetic shift, whereby coinfection can result in influenza viruses exchanging genetic segments to drive the emergence of viruses with dramatically altered genotypes and/or phenotypes. As such, whilst most of the information on AIVs in the literature details outbreaks of disease events involving HPAIV, the circulation and genetic relevance of LPAIVs is also significant. Importantly, LPAIVs often spread silently, causing either very mild clinical disease or being entirely asymptomatic. As such, they are generally only detected where clinical impact involves measurable parameters such as egg drop or comorbidities, which can be exacerbated by intercurrent disease. However, the silent circulation of LPAIVs may contribute to the emergence of viruses with increased virulence as has, for example, been detailed for H7 previously [9], or, where epizootics of HPAIV occur, may drive the emergence of multiple genotypes of HPAIV with donor genes from LPAIVs, potentially impacting on the clinical outcome. Where this is true for H5/H7, it is less clear for other subtypes subject of this case report. Significant knowledge gaps regarding the circulation and genetic characterisation of these LPAIVs exist globally.

Prior to the H6N1 LPAI incursions described in this report, Northern Ireland had not reported AIV infections in poultry since the detection of three cases of H7N7 LPAI in turkeys and chickens in 1997 [10]. This occurrence involved a localised epizootic that extended into Ireland in the same year. Sporadic detections of LPAIVs have been reported during the intervening period, but exclusively in wild bird populations. No spillover events of LPAIVs from wild birds into poultry populations were detected until the LPAIV H6N1 outbreaks detailed here. In 2020, Northern Ireland had a poultry population of more than 24 million birds, 63% of which comprised broilers (DAERA 1981–2020). The economic value of the poultry sector in Northern Ireland increased by 8% to GBP 325 million in 2021, while the egg sector increased by 4% in total value to GBP 125 million (DAERA). As such, the poultry industry is of significant value to the Northern Ireland economy and constitutes the largest private employer in the province. Notifiable avian diseases (NADs) are a major threat to this key industry, particularly outbreaks of AIV. Here, we detail the incursion of an LPAIV H6N1 into 15 commercial premises in Northern Ireland, 13 premises in Ireland, five premises in Scotland, and one in England. We detail the impact of this incursion of an LPAIV into poultry premises spanning four countries, with a focus on the impact across the island of Ireland.

## 2. Materials and Methods

### 2.1. Surveillance and Sample Submission

In Northern Ireland, AIV surveillance is carried out by the Agri-Food & Bioscience Institute (AFBI) at the request of the Department of Agriculture, Environment and Rural Affairs (DAERA). Report cases, where NAD is suspected in poultry and, where animal numbers permit, require the submission of 20 oropharyngeal swabs, 20 cloacal swabs, 20 bloods, and 5 carcasses per epidemiological unit for testing by molecular and serological methods. AFBI also carry out AIV surveillance in wild bird species as part of the Wild Bird Survey (WBS), whereby carcasses of specified avian species are submitted for postmortem and molecular NAD testing. A similar system operates in Great Britain, where the Animal and Plant Health Agency (APHA) test report cases following a similar algorithm, but wild bird surveillance assesses swab material alone for AIVs. NADs detected in Northern Ireland undergo confirmatory testing at APHA as the UK National Reference Laboratory (NRL) and at the Istituto Zooprofilattico Sperimentale delle Venezie (IZSVe), the European Union Reference Laboratory for Avian Influenza and Newcastle Disease.

The Central Veterinary Research Laboratory (CVRL), Virology Division, NRL of Ireland (Department of Agriculture, Food and the Marine (DAFM)), test report cases, following the same algorithm as Northern Ireland, after notification of AIV suspects to the National Disease Control Centre (DAFM). During the H6N1 outbreak, following confirmation of the index case with LPAI infection, 20 carcasses were submitted from each subsequent outbreak for postmortem examination (PME). Tissues (brain, lung, intestine), swabs, and heart blood were then collected from carcasses for analysis.

### 2.2. Virological Investigation

Sampling approaches either focused on oropharyngeal/tracheal and cloacal swabs, with bloods or a range of tissues collected at PME when carcasses were submitted. Tissues collected from carcasses according to local disease investigation protocols, along with oropharyngeal and cloacal swabs, were assessed for the presence of NAD viral RNA using a AIV matrix (M) gene-specific real-time reverse-transcriptase polymerase chain reaction (rRT-PCR) assay [11], in parallel with an avian paramyxovirus type 1 (APMV-1) rRT-PCR assay [12]. Where M-gene PCR-positive samples were detected, the testing triage led to subtype-specific rRT-PCRs to detect the notifiable HA subtypes H5 [13] and H7 [14], and the NA subtype N1 [15]. Those samples shown to be N1 PCR-positive were subsequently tested at APHA using an in-house rRT-PCR H6-specific AIV subtyping assay (available @ fluglobalnet). Briefly, total nucleic acid was extracted from the original clinical material using a QIAmp viral RNA BioRobot kit customised for APHA in conjunction with a Universal BioRobot (QIAGEN, Manchester, UK) [14]. PCR was undertaken for M gene [16] and by H6-specific rRT-PCR, as described previously [17].

### 2.3. Conventional Sequencing and Phylogenetic Analysis

Genetic characterisation of H6 viral RNA following rRT-PCR identification was carried out using Sanger sequencing. The HA cleavage site was amplified and sequenced using specific primers targeting the H6 cleavage site: H6 CS = H6-894f (5′-AGAACTGCGATGCCACATG-3′); H6-1213r (5′-GACCTTATTTGTAATTCCGTC-3′); HA1 (Bm-HA-1; 5′-TATTCGTCTCAGGGAGCAAAAGCAGGGG-3′) and Bm-ns-890r (5′-ATATCGTCTCGTATTAGTAGAAACAAGGGTGTTTT-3′). Partial nucleotide sequence analysis, alignments, and amino acid cleavage site motif analysis were carried out using Lasergene software suite version 10 (DNASTAR, Madison, WI, USA) and the Geneious Prime software suite version 2022.1 (Dotmatics, Boston, MA, USA).

### 2.4. Whole-Genome Sequencing and Phylogenetic Analysis

Virus isolates were obtained from clinical samples using 9- to 11-day-old specified-pathogen-free embryonated fowls’ eggs [7]. Following virus isolation, AIV RNA was extracted manually [14] without the inclusion of carrier RNA. WGS data were generated and assembled as described previously [18].

Consensus sequences of the eight gene segments of strain A/chicken/Northern Ireland/AV30-20-CP3-EP1/2020 (H6N1) were used to query the entire GISAID BLAST database to search for common ancestors from the EPIFlu database on the GISAID website (https://platform.gisaid.org/epi2/ (accessed on 24 June 2024)) with ≤250 alignments. Corresponding gene sequences were downloaded and aligned with the Northern Ireland/Ireland and Great Britain H6N1 sequences using the MAFFT v7 web server (https://mafft.cbrc.jp/alignment/server/ (accessed on 24 June 2024)). Alignments were viewed using Aliview (https://ormbunkar.se/aliview/), with 5′ and 3′ UTR sequences trimmed and sequences with duplicate sample IDs removed. Maximum likelihood phylogenetic trees were obtained for each gene using the IQTREE web server (http://iqtree.cibiv.univie.ac.at/ (accessed on 24 June 2024)), performing ultrafast bootstrap resampling analysis with 1000 iterations and using the best-fitted nucleotide substitution model selected by ModelFinder. Trees were visualised, rooted at midpoint, and annotated using FigTree v1.4.4 (http://tree.bio.ed.ac.uk/software/figtree/), with bootstrap values ≥80 displayed. The resultant trees for each segment are detailed in the Supplementary Materials in Figures S1–S8.

### 2.5. Histopathology and Immunohistochemistry 

Tissue sections were fixed in 10% *v*/*v* suspension in buffered formalin, processed and embedded in paraffin, and stained with haematoxylin and eosin (H&E). Serial tissue sections were also prepared for virus-specific immunohistochemical (IHC) staining to detect influenza A nucleoprotein antigen, as described previously [19]. Histopathology and correlative viral immunohistochemistry of the stained sections were evaluated by veterinary pathologists.

## 3. Results

### 3.1. Emergence of LPAIV H6N1 across Northern Ireland, Great Britain, and the Republic of Ireland

#### 3.1.1. Northern Ireland

**Figure 1:**
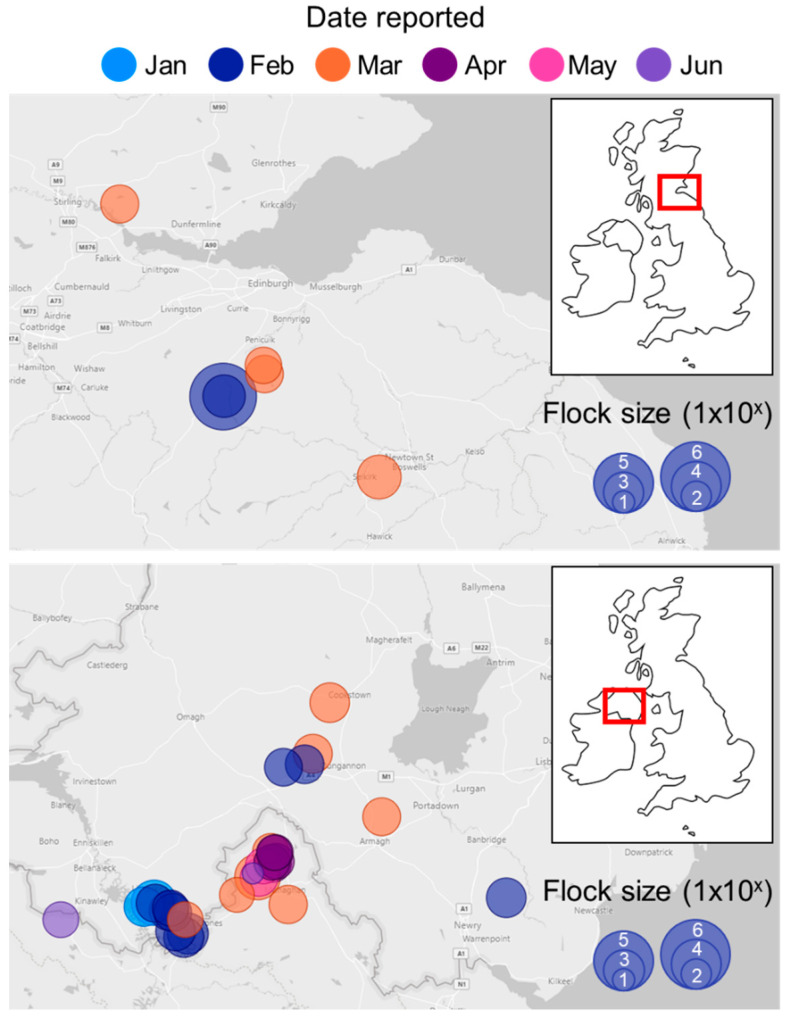
Timeline and location of infected premises across the Republic of Ireland, Northern Ireland, England, and Scotland. Location and flock size of infected premises across affected areas are shown, with the increasing number of premises illustrated at each site. The time of detections is coloured by month according to the key.

The initial detection of LPAIV H6N1 occurred in January 2020 in County (Co.) Fermanagh (Figure 1), located in the south–east of Northern Ireland, which directly borders Ireland, with significant branching of multiple waterways from Upper Lough Erne in Northern Ireland southwards to Ireland. On 3 January, the owner of a broiler breeder flock comprising 28,000 birds reported suspicion of an NAD following rising mortality (Day 0 = 150 deaths; Day 1 = 200 deaths). Although the initial suspicion was suffocation due to the apparent gathering of carcasses in one area of one house at the unit, the private veterinary practitioner (PVP) subsequently referred the case to DAERA and then AFBI as a potential NAD event for further investigation. PME did not reveal any significant abnormalities in the organs or the possibility of systemic infection. Gross observations noted pale, friable livers in all birds; marked congested carcasses but no evidence of petechia or ecchymosis; and only slight congestion in the trachea. Although the lungs were congested, no consolidation was apparent. AFBI tested as per the NAD testing algorithm. Influenza A virus nucleic acid (M gene) was detected by frontline screening rRT-PCR testing, but the samples were negative for H5 and H7 viral nucleic acid. N1 AIV RNA was detected by NA-specific rRT-PCR. Therefore, the virus was declared within Northern Ireland as a non-notifiable HxN1 strain. Samples were shared with APHA for further subtyping characterisation, where the HA-type was determined to be H6 and the NA subtype was confirmed to be N1.

Further submissions followed without detection for two weeks following the initial detection in rural Co. Fermanagh. A large liquid egg producer reported similar issues, including a drop in egg production, increased mortality, and listlessness in the birds. The primary clinical sign was, however, diarrhoea, and H6N1 confirmation followed. This operation was located approximately 4 km from the first IP.

The H6N1 detections were characterised by marked, if not significant, increases in mortality and pronounced diarrhoea and congestion of wattles and combs, with noted nasal discharge. The carcasses remained unremarkable. There was a clear suggestion that systemic disease was not apparent. H6N1 was confirmed following assessment by APHA.

Further detections remained located within Co. Fermanagh. Submissions and detections continued along the same pattern of increased, if not pronounced, spikes in mortality along with reduced egg production in layers or broiler breeders and noted scour. Within these earliest Fermanagh-based cases, PME indicated no evidence of gross lesions in the brain and no marked abnormalities in the joints, heart, lung, liver, spleen, and kidneys. Virus isolation was undertaken in 9-to-11-day-old specified pathogen-free (SPF) embryonated fowls’ eggs at APHA, and DAFM for the Ireland samples, according to international guidelines [7]. APHA undertook an intravenous pathogenicity index (IVPI) on the virus isolated from the pooled brain sample from AFBI case number 2020-002055 (see Table S1) during February 2020, according to internationally agreed guidelines, with a resulting score of 0.00 [7].

By early February 2020, H6N1 had been detected in layers in Co. Tyrone in the west of Northern Ireland, bridging the gap between the Erne waterways and Lough Neagh, around which there is significant high-density poultry production. In the first case in Tyrone, egg production levels had decreased rapidly by up to 50%. Case submissions and detections began to alternate between the two adjacent counties of Fermanagh and Tyrone, with both broiler breeders and layers affected.

These clinical signs continued to be replicated in Northern Ireland, with soft-shelled eggs noted as an addition to the case definition, along with pronounced diarrhoea. Nine IPs were noted in February across three counties, including Co. Down in the Southeast, although not directly adjacent to either Fermanagh or Tyrone. Submissions and detections reduced but continued into March 2020. In addition to egg production drop and soft-shelled eggs, some peritonitis was also noted in these latter cases. The last reported positive detection was in the last week of March 2020. No further H6N1 suspects or detections followed in 2020 in Northern Ireland.

#### 3.1.2. Scotland

Initial disease detection in Scotland occurred on the 5 February 2020, with LPAIV H6N1 detected in a commercial chicken flock in the Scottish Borders after statutory laboratory testing of official samples at APHA-Weybridge. By the 17 March 2020, three further LPAIV H6N1 detections had been made in commercial chicken flocks elsewhere in Scotland, and the fifth outbreak of LPAIV H6N1 in Scotland was confirmed in commercial chicken premises after an initial suspicion of disease on 2 March 2020. The presence of H6N1 in each affected IP was confirmed as described above.

#### 3.1.3. England

The single LPAIV H6N1 case detected in England followed suspicion of NAD in a commercial broiler flock containing 75,000 birds in Shropshire on 28 February 2020, following a massive increase in mortality in two houses from Thursday 27 February 2020. Laboratory confirmation of H6N1 was made, as described above.

#### 3.1.4. Republic of Ireland

In February 2020, a private veterinary surgeon (PVS) reported clinical disease in a table egg layer barn flock located in Co. Monaghan, located 100 metres away from a known LPAI H6N1-positive flock in Northern Ireland. Disease signs included a 34% drop in egg production, which increased to a level of 96% over a 6-day period. In addition, abnormal egg shape and thin shells were reported alongside a small increase in mortality (0.01% to 0.03% over 8 days) and decreased feed and water intake. Tissues and swabs were confirmed positive for H6N1 and negative for H5 and H7 subtypes by molecular methods. Blood serum was AIV antibody-positive on ELISA (IDEXX Influenza A Virus Antibody ELISA) and AGID (Adapted from OIE manual 2021 and IZSVe) but negative on H5 and H7 haemagglutination inhibition tests (Adapted from OIE manual 2021 and IZSVe). Following this confirmation, nine more table egg layer flocks and three turkey fattening flocks were confirmed positive for non-notifiable H6N1. All IPs were in Co. Monaghan, with the last one confirmed on the 19 of June 2020. Of note, a common clinical sign observed in the layers was watery diarrhoea with occasional green colouration. In fattening turkeys, lethargy and low-level mortalities (<2%) were observed.

### 3.2. Impact of Infection and Clinical Disease Assessments

#### 3.2.1. Clinical Disease

As the outbreak progressed in Northern Ireland and Ireland, the decision was taken by the competent authorities to rapidly define common clinical signs to distinguish a potential H6 suspect case for investigation. Based on information collected via the triaging of cases, the following clinical signs were used as an indicator of infection: reduced feed and water intake; behavioural changes such as dullness and depression; egg drop occurring suddenly (<48 h) or gradually (e.g., over 7 days), with an increase in the number of soft-shelled, discoloured eggs (pale to white-shelled eggs in brown egg layers); watery diarrhoea with or without green colouration; a small increase in mortality (<2%). Alongside these, broiler breeder flocks appeared to suffer rooster mortalities along with ocular signs and changes in the combs. In Ireland, flocks located within the counties of Cavan or Monaghan, or with an epidemiological link to a confirmed case and one of the clinical signs above, would meet the H6 LPAI case definition.

#### 3.2.2. Gross and Microscopic Pathological Observations

**Figure 2:**
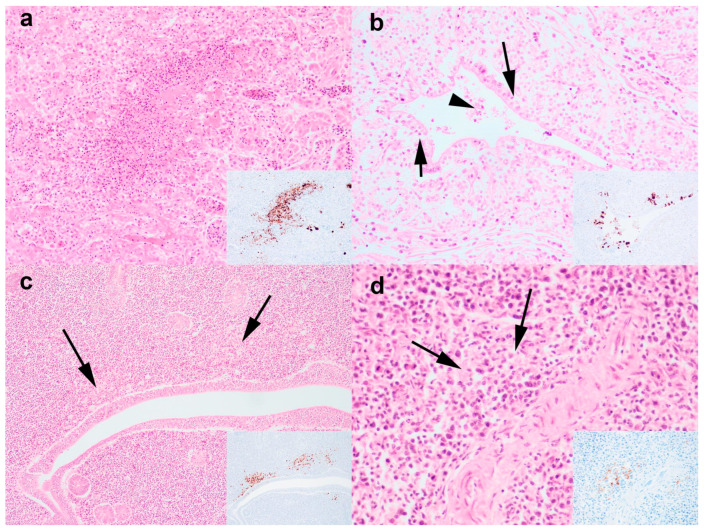
Histopathological changes associated with avian influenza virus infection. Severe necrotising tubulointerstitial nephritis (**a**); mild suppurative salpingitis (arrow refers to intra-epithelial heterophils and arrowhead refers to exudate and cellular debris (**b**); mild lymphoid depletion (arrow) in the spleen (**c**); and caecal tonsil (**d**). Insets are immunohistochemical-labelled serial tissue sections imaged at the same magnification. Serial sections are stained with haematoxylin and eosin and influenza A nucleoprotein antibody (insets). Original magnifications 100× (**c**), 200× (**a**,**d**), 400× (**b**).

**Figure 3:**
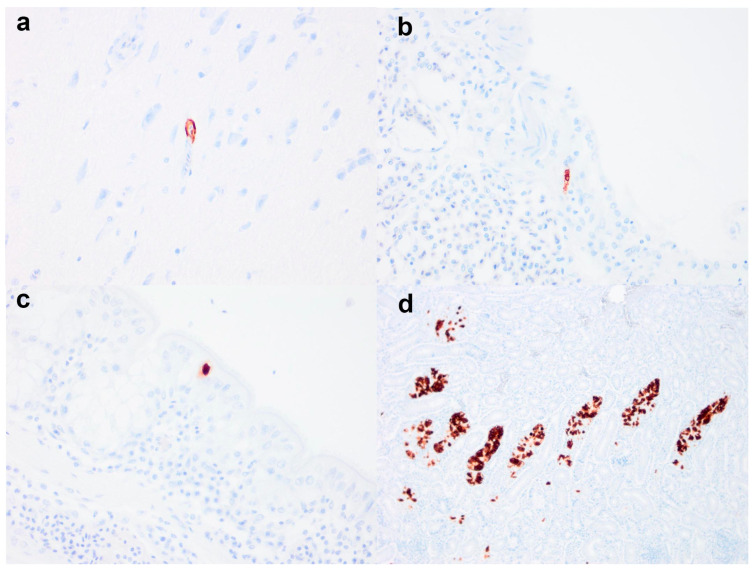
Immunohistochemical detection of influenza A virus antigen. Rare vascular labelling was detected in the cerebral capillary (**a**) and air capillary (**b**). Rare and moderate levels of epithelial immunolabelling were detected in the tracheal (**c**) and renal tubules (**d**). Sections were immunolabelled with influenza A nucleoprotein antibody. Original magnifications 100× (**d**) and 400× (**a**–**c**).

Necropsies conducted at APHA (n = 14) for Scottish and English cases revealed non-specific changes in the carcasses, including coelomitis (n = 4), oophoritis (n = 3), salpingitis (n = 3), visceral gout (n = 2), splenomegaly (n = 4), and renomegaly (n = 3). Viral IHC performed on the birds (n = 14) revealed the presence of viral antigens in the brain (n = 1/12), spleen (n = 6/13), kidney (n = 5/14), caecal tonsil (n = 1/8), nasal turbinate (n = 1/12), trachea (n = 1/13), lung (n = 1/13), oviduct (n = 2/6), and ovary (n = 1/9). Specifically, viral antigens were localised in the vascular endothelial cells of the brain and lung (Figure 2a,b; same bird); epithelial cells of the nasal turbinates, trachea (Figure 2c), renal tubules (Figure 2d), and caecal tonsil; and lymphoid cells in the spleen and caecal tonsil. Viral antigens were generally rare; however, in the kidney, a moderate amount of viral antigen was detected. In cases where viral antigens were detected, correlative viral IHC and histopathology analyses revealed pathology associated with AIV infection, including mild tubular epithelial degeneration (n = 1/5) or moderate-to-severe necrotising tubulointerstitial nephritis (n = 4/5; Figure 3a); mild suppurative salpingitis (n = 1/2; Figure 3b); mild lymphoid depletion in the caecal tonsil (n = 1/1; Figure 3c) and spleen (n = 2/6; Figure 3d); mild lymphocytic tracheitis (n = 1/1) and rhinitis (n = 1/1). Other background infection and lesions detected include tracheitis with intralesional intranuclear inclusion bodies (n = 1/13; infectious laryngotracheitis), intestinal nematodiasis (n = 1/13), and intestinal coccidiosis (n = 3/13). Other non-specific histopathological findings included visceral gout-associated myonecrosis (n = 1/12) and air capillary necrosis (n = 2/12), lymphocytic rhinitis (n = 1/11), and splenic lymphoid hyperplasia (n = 4/13).

With the Ireland cases, following the PME of layer hens, no or partially formed eggs in oviducts were observed, but active follicles and suppurative peritonitis were found in some cases. Some turkeys showed marked pulmonary congestion and oedema in viscera.

### 3.3. Genetic Evaluation of LPAIV H6N1 from Outbreak Events

Phylogenetic analyses were performed using the sequences obtained from Northern Ireland/Ireland, and Great Britain isolates, along with other relevant sequences, including those with the highest similarity to the Irish sequences. The degree of sequence identity shared between viruses from the island of Ireland was high across all eight segments, ranging from 98.9 to 100% (Table 1). Analysis of the HA, NA, and PB2 genes demonstrated that the H6N1 sequences from Northern Ireland, Ireland, and Great Britain formed distinct clades (Figures S1–S3), whilst the Northern Ireland and Ireland HA, NA, and PB2 sequences demonstrated high degrees of sequence identity (99.2–100%). The sequences from Great Britain formed separate sub-clades and shared lower degrees of sequence identity (95.7–98.9). The PB1, PA, NP, MP, and NS segments of the Great Britain viruses were distinct from the Northern Ireland/Ireland sequences, with correspondingly low sequence identities ranging from 93.3 to 97.5% (Figures S4–S8 and Table 1). Taken together, these data indicate that the Great Britain H6N1 strains form a distinct genotype whose HA, NA, and PB2 segments share a common origin with the Irish sequences and suggest that the Irish and Great Britain outbreaks represent separate introductions. For the Irish isolates, most segments are derived from a common pool of Eurasian LPAIVs of various subtypes, with the exception of the PB2 segment, which is related to those found in HPAIV H5N1. All viruses isolated in this study were demonstrated to have the HA cleavage site motif PQIETRGLF or PQLETRGLF, both consistent with LPAIVs and with the demonstrated IVPI score of 0.00 [7].

In Ireland, 165 wild birds were screened for AIV by PCR during this period, with 23 confirmed as H5N8 HPAI-positive, but none were H6N1 LPAIV-positive. However, the surveillance schemes across different areas rely on mortalities or symptomatic infection and, as such, only AIV strains causing mortality would be detected. This leaves a gap in which LPAIVs that are unlikely to cause clinical disease in non-poultry species would not be detected. No cases of H6N1 LPAIV were detected in similar surveillance schemes from across Scotland, England, and Northern Ireland during the same time period.

## 4. Discussion

Incursions of NAD, including both HPAIV and Newcastle Disease Virus (NDV), are globally the primary concern across both wild bird and poultry populations. The ongoing epizootic of clade 2.3.4.4b H5Nx HPAIV has vividly demonstrated the impact that HPAIVs can have within poultry settings and across wild bird populations [20,21,22,23,24,25]. LPAIVs are of high significance to the poultry industry, as these viruses can circulate asymptomatically in different avian species and can have a significant impact upon poultry health and productivity at the same time. H6Nx has become an increasing burden on the poultry industry worldwide [26] and is essentially considered to be enzootic in Asia [27], easily transitioning from live bird markets into domestic poultry. H6Nx is problematic in that it notably has the most extensive host range when compared to other AIV subtypes [28]. H6 viruses have been associated with several orders of wild birds (especially anseriformes) but have also spread to gallinaceous poultry including pheasants, with evidence suggesting that geese might be the main migratory vector for H6Nx influenza viruses [27]. Although well characterised, with the earliest known sequence dating to 1963 from turkeys in Canada [26,29,30], H6Nx AIVs remain a persistent production threat, with the frequency of infections on the rise across Europe and some suggestion that they may be increasingly poultry-adapted [27].

In the UK, circulation of LPAIVs are sporadically encountered, usually as a result of mild clinical signs, but are excluded using a differential testing algorithm under programmes such as the ‘testing to exclude’ scheme [31]. On occasion, more overt signs are reported, which trigger a formal veterinary investigation and precautionary sampling and testing to exclude LP H5 and H7 viruses. In addition, official serological surveys of poultry premises returning influenza A antibody tests can elicit formal investigation and further sampling. Under both surveillance strands, non-negative surveillance results are followed up by official statutory NAD investigations of the suspect IPs. Northern Ireland does not have a corresponding ‘testing to exclude’ policy. Symptomatic poultry are tested as part of epizootic testing in response to a suspected NAD. LPAIVs can act as donors for virus segment exchange and the emergence of novel viruses following coinfection and genetic reassortment, while LPAIVs of subtype H5 or H7 can potentially lead to the emergence of HPAIV H5 or H7 through LP-HP mutation events [6]. Further, LPAIVs of different subtypes have also been associated with zoonotic events (e.g., H5, H3, H7, H9, H10, and H6) [32,33,34,35] that are reportable to WOAH when an animal source is identified. Globally, the detection of H6 viruses in poultry settings continue to be sporadic. The role of potential co-infection with other pathogens may exacerbate the outcome of infection with LPAIVs, and the role of concomitant infection driving differential disease outcomes remains a significant knowledge gap. H6N1 infection has been linked to co-infection in a case in turkeys in France, whereby H6N1 was detected alongside pronounced bacterial congestion [36], although defining causative agents for disease manifestation remains challenging. However, regardless of the subtypes in circulation, these LPAIVs may go undiagnosed as they may cause little-to-no disease or may be the agent that drives immune dysregulation, laying a path for secondary bacterial infections. Detection and recording of H6N1 and other LPAIVs remain important, especially in regions dependent on a densely stocked poultry sector. There have been noted cases in turkeys in Israel, with several H6 identifications since 2000 [37]. However, except for occasional reports in poultry, H6Nx had only been reported previously in pheasant and quail flocks [38]. Interestingly, since this detection, novel H6 reassortments have been detailed in chickens in live poultry markets in China [2].

In the current report, the development of disseminated infection and associated lesions with defined clinical disease outcomes is important in defining the potential impact of these viruses. The clinical presentation described in this series of cases, alongside pathological observations of viral dissemination more readily associated with HPAIV infection, again supports the need to characterise the mechanism of localised and multiorgan LPAIV infections in poultry. Given the conventional understanding of the epitheliotropism of LPAIVs, the observation of viral antigens (albeit in a low proportion of examined birds) in both vascular and neurological tissues, in our cases, are unusual characteristics for LPAIVs. 

Alongside their potential impact on poultry production and wild bird health, LPAIs represent both a reservoir for potential reassortment and emergence of novel pathogens as well as an invasive threat through spontaneous mutation. Regardless, the asymptomatic circulation of LPAIVs is a pertinent example of the need to always maintain effective biosecurity. Mutations within the HA gene are frequent, with the well-characterised E190V and G228S mutations and recurrent reassortment demonstrating the malleability of H6Nx AIVs [39]. Such genetic plasticity may account for the formation of sub-clades, where those detected on the Island of Ireland are similar across many genes when compared to those occurrences in Great Britain. Temporally, LPAI detections in poultry occurred in Northern Ireland, followed by Great Britain and then Ireland. Figures S1–S8 illustrate the somewhat divergent nature of the H6N1s when contrasting those detected on the Island of Ireland with those detected in the northern-most parts of Great Britain. Reassortment between various HA/NA subtypes and internal protein genes derived from multiple lineages occurs widely in nature amongst numerous wild bird species. Considering the genetics, we can speculate that there may have been multiple primary introductions from wild birds, but some secondary spread must be plausible. Secondary spread via contaminated clothing, equipment, or vehicles is possible; however, the epidemiological linkages between IPs have not been fully determined. It appears highly unlikely that that all incursions across a wide geographic area were due to high incidence of H6 in wild bird species. However, long branch lengths in the eight phylogenies that separate geographical clusters from the most closely related sequences must be treated with caution, since the lack of data is indicative of gaps in the genomic surveillance of influenza A viruses circulating in wild birds. This lack of data prevents an accurate determination of the precise origins and diffusion of these strains. Furthermore, although the HA, NA, and PB2 segments demonstrate a common origin between Great Britain and Irish viruses, the Great Britain cases are not the same virus as those identified on the Island of Ireland. The linkage between Great Britain farms and those on the Island of Ireland remains very unlikely. Whether this precludes a single reservoir of origin for incursions across both islands remains open for debate. While it cannot be ruled out, it would seem less likely that two LPAI incursions occurred in relative proximity from alternate sources.

LPAIVs’ environmental persistence can act as a bridge to infections of greater severity [40]. Although much of the focus remains on notifiable AIVs, ultimately H5 and H7, the presence of other LPAIV subtypes represent a risk of LPAIV to HPAIV transition and the possibility of an emergent threat not being recognised immediately. In 2002, a turkey farm in the Netherlands tested positive for an H7N3 LPAIV, where there were no immediate indications of impactful clinical disease observed. Several years later, deep sequencing suggested a minority of HPAIV H7 sequences (0.06%) were present [41]. The minority HPAIV variant exhibited a 12-nucleotide, single-event insertion in the HA cleavage site. Experimentally, the HPAIV H7N3 rapidly outcompeted the LPAIV H7N3, suggesting a near-miss in the emergence of a HPAIV. It is not clear why LPAIV H7N3 was maintained as a dominant population in the field. Further, Le et al. [42] recently noted the threat of LPAIVs, using Muscovy ducks in Vietnam as an example where the estimation of flock impact is difficult to quantify, especially where recovery in those populations occurs. Again, these reports substantiate that complacency in biosecurity is more readily assessed where a HPAIV emerges and causes considerable mortalities. Non-H5/H7 LPAIVs can have a large impact on poultry production, and this is compounded by the fact that, technically, government intervention is not mandated.

In an isolated turkey breeder unit in the UK (~5000 birds), a H9N2 was detected after the rapid onset of symptoms and morbidity escalating from 10% to 90% in a short period. With an intravenous pathogenicity index of 0.00, no official control measures were applicable. Although the morbidity was high, many birds recovered and were put back into production [43]. This raises the concern that multiple LPAI incursions are ongoing at any one time. In the case of the outbreak detailed here, many infected premises in Northern Ireland chose depopulation over resolving the infection to maintain confidence in the industry and prevent onward spread. In many cases, this meant the culling of birds in the 100s of 1000s range in efforts to take control of the situation in a geographically small region with a very high poultry-stocking density. Given the number of confirmed cases and presumed infectivity of the H6N1 viruses for domestic poultry, without such industry action the scale of the event could have been much greater.

From a risk perspective for incursion of AIV in Northern Ireland, the province has extensive wetlands hosting migratory birds travelling along the East Atlantic Flyway. These areas extend to Lough Neagh, the largest (by area) freshwater lake in the British Isles, as well as Upper Lough Erne and Lower Lough Erne, both in Co. Fermanagh, in the southwest of Northern Ireland. Upper Lough Erne branches into a furcating network of smaller waterways that continue southward into Co. Cavan in Ireland. Co. Cavan is bordered to the east by Co. Monaghan, which is surrounded on three sides by the Northern Ireland counties Fermanagh, Tyrone, and Armagh, with Counties Tyrone and Fermanagh being the epicentre for poultry and egg production in Northern Ireland. The LPAI H6N1 outbreak in Northern Ireland was a timely reminder of the continual need to exercise rigorous biosecurity.

From an H6 perspective, elevated microneutralization antibody titres to A/Teal/Hong Kong/w312/1997 (H6N1) had previously been noted in a small number of Romanian agricultural workers, but none of these workers had met a pre-set criteria for potential exposure from a poultry source [44]. China has long-recognised H6 subtypes as commonly occurring in domestic ducks. In 1997, an H6N1 isolated from a teal in Southern China was at the time believed to be a potential progenitor of A/Hong Kong/156/1997 (HK/97) H5N1 virus because seven of the eight gene segments appeared to share a common source [38]. Of further interest is the detection of H6N1 in other mammalian species. H6N1 has recently been detected in domestic dogs in Taiwan. Upon genetic evaluation, this Taiwanese detection was determined to carry an E627K substitution in the PB2 gene, a mutation associated with adaptation to mammalian hosts and it, as such, may represent increased zoonotic risk, especially where infection of companion animals is concerned [45].

In conclusion, H6N1 LPAIVs affected multiple premises across four countries, causing significant losses to industry in those areas. A wild bird source was not defined, yet it is presumed that the virus most likely derived from a wild bird source that caused at least a proportion of the confirmed cases. The lack of pre-outbreak evidence could suggest the long-term persistence of asymptomatic H6N1 in galliform populations. The subsequent emergence of a more infectious variant to cause these outbreaks cannot be excluded per sé, but had this been true it is likely that more cases would have been observed and reported. This group of H6N1 viruses were infectious for domestic birds, causing clinical effect without apparent adaptation, and were able to spread. Industry action across all affected regions on both islands likely mitigated the economic impact. The impact was not insignificant for affected producers, with many taking the decision to depopulate all houses in their operations. Genetic analyses support unrelated introductions between the island of Ireland and Great Britain, and as such the characterisation of LPAIVs is a significant gap in our knowledge of AIVs. This lack of knowledge is worsened by the fact that there are few reports of LPAI outbreaks in Europe and other parts of the world. Screening of both wild bird and poultry populations, in-depth characterisation of all identified AIVs, and reporting of any findings is the only way that knowledge in this area will be improved.

## Figures and Tables

**Table 1:** Percentage nucleotide identity shared between Northern Ireland and Ireland/Great Britain sequences.

	HA	NA	PB2	PB1	PA	NP	MP	NS
Ireland	99.2–99.9	99.2–99.9	99.4–100	99.5–99.0	99.5–99.0	99.1–99.8	99.1–100	98.9–100
Great Britain	95.7–96.0	98.0–98.5	98.5–98.9	93.4–93.8	94.6–95.1	93.3–93.8	97.2–97.5	94.9–95.4

## Data Availability

Sequence data generated in this study have been uploaded to GISAID (https://www.gisaid.org) under isolate IDs EPI_ISL_2234825 to EPI_ISL_2234830.

## Supplementary Materials

The following supporting information can be downloaded at: https://www.mdpi.com/article/10.3390/v16071147/s1, Table S1: Case information for IPs from Northern Ireland, Republic of Ireland, and Great Britain. Figure S1: Maximum Likelihood tree comparing HA of Northern Ireland/Republic of Ireland H6N1 with related sequences. Figure S2: Maximum Likelihood tree comparing NA of Northern Ireland/Republic of Ireland H6N1 with related sequences. Figure S3: Maximum Likelihood tree comparing PB2 of Northern Ireland/Republic of Ireland H6N1 with related sequences. Figure S4: Maximum Likelihood tree comparing PB1 of Northern Ireland/Republic of Ireland H6N1 with related sequences. Figure S5: Maximum Likelihood tree comparing PA of Northern Ireland/Republic of Ireland H6N1 with related sequences. Figure S6: Maximum Likelihood tree comparing NP of Northern Ireland/Republic of Ireland H6N1 with related sequences. Figure S7: Maximum Likelihood tree comparing MP of Northern Ireland/Republic of Ireland H6N1 with related sequences. Figure S8: Maximum Likelihood tree comparing NS of Northern Ireland/Republic of Ireland H6N1 with related sequences.
