# Supplementary material for: Evaluating the Impact of Low-Pathogenicity Avian Influenza H6N1 Outbreaks in United Kingdom and Republic of Ireland Poultry Farms during 2020
Source: Viruses. 2024 Jul 16;16(7):1147. doi: 10.3390/v16071147 (PMC11281592; doi:10.3390/v16071147)
Supplement: Supplementary file 1 [file viruses-16-01147-s001.zip › Table S1.pdf]

Table S1: Case information for IPs from Northern Ireland, Republic of Ireland and Great Britain

Date received	Flock category	Country	Location	Flock size	Unique identifier	Testing outcome	Case / PM Notes / History / Necropsy / Conclusions
03/01/2020	Broiler Breeders	Northern Ireland	Co. Fermanagh	28,000	2020-000058	*H6N1*	Flock owner reported increased mortality in a broiler rearing premises with mortality limited initially to one section of a house. Smothering was considered as cause of mortalities but concerns of NAD raised. PME demonstrated pale friable livers and congested carcasses although no evidence of petechiation or ecchymosis. Trachea were slightly congested. No significant abnormalities detected in proventriculus, intestine and gizzard. Lungs were congested but not consolidated. Spleens not enlarged. Kidneys pale, slightly enlarged.
17/01/2020	Layers	Northern Ireland	Co. Fermanagh	375,000	2020-001067	*HxN1*	A PVP reported a commercial egg laying flock presenting with diarrhoea and listlessness. On farm PME gave evidence of petechiation in the proventriculus. NAD suspected and PME at AFBI detected multifocal hepatocellular necrosis (central area of necrotic cells, degenerate granulocytes bordered by macrophages, multinucleate giant cells and fibrin surrounded by an outer rim of lymphohistiocytes) in the liver, intestinal focally extensive granulocytic enteritis with transmural inflammation extending to fibrinous serositis. Occasional glands were dilated with degenerate granulocytes and basophilic debris.
03/02/2020	Broiler Breeders	Northern Ireland	Co. Fermanagh	16,000	2020-002055	*HxN1*	One house out of three houses affected. Increasing mortality, extensive diarrhoea. Post-mortem at AFBI – Birds exhibited congestion of wattles and comb with varying degrees of serous nasal discharge. Crops were mostly full with seed material, one crop having a brown, watery content. Skin was dark with subcutaneous congestion and darkened appearance to muscle tissue. Mucous present within beaks of all post-mortemed birds with varying degrees of congestion of tracheas, some with a thick, mucous content. Lungs appeared slightly congested with occasional haemorrhage. Heart muscles appeared dark. All birds gad a pale, greasy appearance to livers. Spleens were generally unremarkable but kidneys of all birds had petechial haemorrhage over surface. Mesenteries were congested and gut content ranged from normal to brown liquid, with 2 having haemorrhagic content with prominent Peyer's patches. All gizzards were packed with seed content and some mucoid layer over proventricular serosal surface. 3 of 4 birds had pinpoint haemorrhage within proventriculus.
03/02/2020	Layers	Northern Ireland	Co. Fermanagh	7,000	2020-002146	*HxN1*	A private veterinary practitioner reported illness in a commercial egg flock. The main clinical signs included a slightly increased mortality, reduced egg production, and scour in some of the birds. 2 houses on site. A post-mortem on farm revealed petechiae on the proventriculus in 2 out of the 8. There were little other clinical signs.<br>Post-mortem at AFBI – Gross examination unremarkable, all birds in lay. 1/5 birds showed Salpingitis/egg peritonitis. No gross lesions consistent with severe systemic disease. No significant abnormalities detected in brain, SC, muscles, joints, heart, lungs, liver, spleen, kidneys, and gastrointestinal/alimentary tract.
03/02/2020	Layers	Northern Ireland	Co. Tyrone	14,000	2020-002145	*HxN1*	A private veterinary practitioner reported a reduction in egg production to 4,000 eggs (instead of the expected 8,000) in one of the houses of a free-range commercial laying flock. The birds had been put on antibiotics but there was no response. The other house is unaffected.<br>All birds in lay, unremarkable on gross examination with no lesion consistent with severe systemic disease. Large intestine contents watery but serosa and mucosa unremarkable. Small intestine contents watery but serosa and mucosa unremarkable.

03/02/2020	Broiler Breeders	Northern Ireland	Co. Fermanagh	14,600	2020-002215	*HxN1*	A private veterinary practitioner reported clinical signs in a broiler breeder unit, similar to those found in poultry at one of the previous H6N1 sites. This is a broiler breeder flock at approximately 59wks of age. The site has been restricted, samples have been taken and a veterinary investigation is ongoing.<br>Post-mortem at AFBI - Submitted carcasses showed lesions consistent with systemic disease as well as other incidental conditions. The wattles were reddened and swollen on external examination. In 2/5 birds there was thickening of the pericardium associated with the presence of fibrin clot in the thoracic cavity and cloudiness of the air sacs. In all cases the lungs were hyperaemic and oedematous. The liver and spleen were congested in each case; in one bird the visceral surface showed a fine, white, granular deposit consistent with visceral gout. Kidneys were pale and mottled in each case. In 2 cases there was ecchymotic haemorrhage of the proventriculus with mucosal necrosis; in all cases the small intestine and large intestine contents were pale-green and watery. One bird had ascites.
04/02/2020	Layers	Northern Ireland	Co. Tyrone	30,000	2020-002238	*HxN1*	A private veterinary practitioner reported egg drop in 1 house of a free-range layer commercial premises. A 2nd house, a barn unit, appeared normal at time of reporting.Post-mortem at AFBI - The birds did not show gross lesions consistent with systemic disease. 2/5 birds showed salpingitis/"egg peritonitis" and in each bird the liver appeared fatty and the abdominal cavity contained a large amount of yellow fat. The livers were friable, consistent with fatty liver. 2/5 birds had salpingitis/"egg peritonitis" with inspissated yolk material present in the abdominal cavity.
05/02/2020	Commercia l layers	Scotland	Scottish Borders	320,000	DPR2020/02	*H6N1*	A private veterinary surgeon reported suspicion of Notifiable Avian Disease in a commercial layers unit (table eggs). 1 of 10 houses affected (around 32000 birds per house). Egg production significantly dropped; mortality has increased to 70 birds per day; reduced water and feed intake and wet droppings. The layers did not improve after antibiotic treatment.<br>APHA vet investigated and confirmed PVS findings. Confirmed also that these are free range birds. Samples were negative by serology for H5, H7 and Newcastle Disease and by PCR for Newcastle Disease, H5 and H7. Virus was isolated from eggs at day 2 and subtyped as H6. Preliminary data indicates that the virus is of a low pathogenicity strain (LPAI).<br>PME (n=2) at APHA found coelomitis (n=1), salpingitis (n=1) and splenomegaly (n=1)
06/02/2020	Layers	Northern Ireland	Co. Tyrone	40,000	2020-002500	*HxN1*	A private veterinary practitioner reported a free range commercial laying site. 4 houses on site, with only one house showing clinical signs at time of report. The main clinical signs are soft-shelled eggs, decreased production and diarrhoea.<br>Post-mortem at AFBI - 5/5 congested carcasses with egg peritonitis. Occasional subcutaneous petechiae in neck region. Mucus in nasal passages. Some pale faecal soiling at vent. Oviductal vessels congested and with occasional petechiae. 2/5 spleen rather enlarged with multiple pale foci.
13/02/2020	Broiler Breeders	Northern Ireland	Co. Tyrone	21,000	2020-002754	*HxN1*	A private veterinary practitioner reported a broiler breeder unit. The clinical signs reported were similar to other broiler breeder cases including dead roosters, mildly swollen liver/spleen. Petechial haemorrhages in muscle and proventriculus and swollen eyelids.
22/02/2020	Layers	Northern Ireland	Co. Down	32,000	2020-003395	*HxN1*	A private veterinary practitioner reported a suspect case in a commercial laying unit in Co. Down. The premises consists of 2 commercial egg layer houses, 16,000 birds each. Clinical signs included reduced feed intake, reduced egg production, with the production of white eggs in a brown egg producing unit.

							Post-mortem at AFBI - Kidneys pale and mottled; livers pale and friable, consistent with fatty infiltration; large intestine contents were watery; ovaries exhibited moderate to severe suplingitis / egg peritonitis was present in each case & small intestine contents were watery.
22/02/2020	Layers	Northern Ireland	Co. Fermanagh	31,000	2020-003394	*HxN1*	A private veterinary practitioner reported a suspect case in a commercial laying unit in Co. Fermanagh. This site is close to another affected premise. It was a free range commercial egg producer with 2 houses, with 31,000 birds on site. The clinical signs included decreased egg production, decreased feed intake and soft shelled eggs.
26/02/2020	Commercia l layers	Scotland	Scottish Borders	320,000	DPR2020/04	*H6N1*	A private veterinary surgeon reported suspicion of Notifiable Avian Disease in commercial layers (table eggs). This is the same premises previously investigated as DPR 2020 02 (where another house (H2) was affected). H1 subsequently showed increase in mortality and significant drop in egg production (from 90% to approx. 10%). APHA vet investigated and was able to confirm the clinical signs and mortality figures initially reported by the PVS.
							PCR for H5, H7 and APMV1 (L gene) and Serology for H5 and H7 are all negative. Serology for Newcastle Disease is positive (birds have been vaccinated for Newcastle Disease). PCR for influenza A (M gene) is positive. Virus isolation results on day 2 are negative (no haemagglutinating virus isolated at 2 days). Subtyping sequencing confirmed this virus was low pathogenicity and characterised as H6N1. The remaining eggs at day 6 Virus Isolation were negative. The suspicion of Notifiable Avian Disease was therefore negated.
							PME (n=4) at APHA found coelomitis (n=2), oophoritis (n=2) and salpingitis (n=1)
28/02/2020	Free Range Table egg layers	RoI	Co. Monaghan	58,500	Dubl/20/00101	*H6N1*	HISTORY: Table egg layers, 980 cages in the house Egg drop also noted. See also PVir/20/00153 Reported by PVP. The farm is located less than 1km from NI where LPAI has been confirmed. Enriched cage system house. Populated with 58,500 birds. Approximate mortality 500. Birds are 49 weeks of age.<br>Mortality: Flock Ownerreports that it was normal on Thursday (6 birds), Friday and Saturday (10 birds each day) but 20 birds dead on Sunday and 27 this morning.<br>Feed consumption: normal up until Saturday: average/bird - Thur 133gms, Friday 100gms, Saturday and Sunday 90gms. No figure for today yet but suspect it to be below average of 117gms/day.<br>Water intake decreased but not as dramatically as the feed intake.<br>Egg production: Friday 51,840, Saturday 34,560, Sunday 25,920 and today 17,280.<br>Ventilation : house is naturally ventilated with side vents and outer access via the roof.<br>Flock Owner noticed marked decreased feed consumption on Sat morning with reduced egg production and whiter eggs at the back of the house. The manure is removed weekly on Monday from the house. It appears wetter.<br>LABORATORY TEST RESULTS:<br>Samples have tested positive for AI MP and H6 N1 PCR. Borderline positive in brain, High positive in the rest. Samples are negative for H5, H7.<br>Virus isolation was carried out on 4 samples and Haemagglutinin (HA) fragment sequenced.<br>DIAGNOSIS & COMMENT:<br>LP AI H6N1. Based on HA fragment, the sequence differs from 2 base pairs to that one found in a close flock from Northern Ireland. Both strains share more than 99% similarity.

28/02/2020	Broilers	England	Shropshire	75,000	DPR2020/05	*H6N1*	A private veterinary surgeon reported suspicion of avian notifiable disease in 33 D.O. broiler chickens. House 4 (23,000 birds) 30% mortality over the last few hours, 60% morbidity with respiratory signs, malaise, congested combs and wattles and purple patches on legs. House 3 (18,000 birds) thousands dead (not yet counted), similar clinical signs and morbidity to house 4. APHA vet investigated and confirmed findings. Birds were lethargic and gasping for air before death. Notifiable Avian Disease could not be ruled out, so samples were taken. PCR for H5, H7 and APMV1 (L gene) and Serology for H5, H7 and Newcastle Disease were all negative. PCR for influenza A (M gene) was positive. Day 6 virus isolation was positive. Laboratory confirmed it as being H6N1 with a Low Pathogenic AI motif by PCR.<br><br>PME (n=4) at APHA found splenomegaly (n=1) and pododermatitis (n=4)
02/03/2020	Commercia l layers	Scotland	Scottish Borders	600,000	DPR2020/07	*H6N1*	Suspicion of avian notifiable disease was reported on a large commercial layer unit in Scotland. This premises is part of the same group of farms linked to DPR 2020 04. Birds in four of six sheds (two are unstocked) exhibited a 10% drop in egg production, increased mortality and reduction in feed and water intake. The premises also housed an egg packing station which received eggs from another free range unit nearby.<br><br>Preliminary PCR results were Negative for AI H5 and H7, and Newcastle Disease V (L gene) in all houses, but Positive for M gene indicating an Influenza A virus in Houses 4,5 and 6. Haemagglutinating virus has been isolated after 2 days incubation. Subtype sequencing indicates this virus to be of Low pathogenicity, H6N1.<br><br>PME (n=2) at APHA found systemic gout (n=1), ovarian regression (n=2), salpingitis (n=1), splenomegaly (n=1) and renomegaly (n=1)
04/03/2020	Broilers	Scotland	Clackmannanshi re	42,000	DPR2020/08	*H10N4*	On Monday 2.3.2020, PVS was granted approval for Testing to Exclude (TTE) on this Broiler breeder farm where egg production had dropped by 5% over the weekend. PVS visited and reported only a slight drop in food consumption and pale eggs; no other clinical signs of Notifiable Avian Disease was suspected. Samples for bacterial and mycoplasma disease screening were taken and in view of current cases of Low Path AI, TTE was requested. All egg movements from the farm have been self-restricted pending TTE results. APHA Weybridge reported results indicating M gene presence and so official investigation with sampling was carried out. PCR results were negative for H5, H7 and Newcastle Disease, positive for Influenza A. Haemagglutinating virus isolated @2days. Subtype sequencing indicates this virus to be of Low pathogenicity, H10N4.<br><br>PME (n=2) at APHA found systemic gout (n=1), splenomegaly (n=1) and renomegaly (n=2)
04/03/2020	Layers	Northern Ireland	Co. Tyrone	23,000	2020-004049	*HxN1*	A private veterinary practitioner reported suspicion in Co. Tyrone in a commercial egg laying flock, with one of 5 houses on site is affected. The main clinical signs included significant egg drop, and the presence of misshapen, and soft shelled eggs. There was low mortality.<br><br>Post-mortem at AFBI - All birds showed a degree of peritonitis with lesions ranging from mild to moderate; there were no gross changes consistent with systemic disease.

04/03/2020	Turkeys	RoI	Co. Monaghan	450,000	Dubl/20/00153	*H6N1*	HISTORY: 5 X Turkeys, mortality & birds quiet<br>H6N1 positive tested in PVP samples<br>See also Dubl/20/00124 and PVir/20/00191<br>GROSS NECROPSY:<br>Saw dust liver in 3 birds, airsacculitis in one, peritonitis in another.<br>LABORATORY TEST RESULTS: H6N1 LPAI
10/03/2020	Layers	Northern Ireland	Co. Tyrone	32,000	2020-004603	*H6N1*	A private veterinary practitioner reported a commercial egg laying flock, 3 house caged unit; birds 60 weeks old. Flock keeper noticed a small egg drop about 2-3 weeks before reporting problems in their first house; this spread to a second house with a similar egg drop. Mortality was minimal and birds were healthy. However it spread continued to a third house, the egg drop was significant, with a drop to 40% (the others were at 80%), with soft shell and jelly eggs. The birds were also dull, depressed. There is no diarrhoea.
10/03/2020	Laying Hens	RoI	Co. Monaghan	40,000	Dubl/20/00140	*H6N1*	HISTORY: HOUSE 2: 40000 laying hens on site. One house of 12000 affected- egg drop from 80 to 70 % since yesterday, green watery diarrhoea, small increase in mortality, decreased feed in-take (120g to 104gr). Slight depression in birds but not remarkable. Two other houses affected with green stained eggs. 5 birds PTS from Hse 3 .<br>GROSS NECROPSY: Green diarrhoea in one bird, peritonitis in 2 birds<br>LABORATORY TEST RESULTS: H6N1 LP strain<br>Conclusion: H6N1 LP strain in House 2.
12/03/2020	Hens	RoI	Co. Monaghan	18,000	Dubl/20/00152	*H6N1*	HISTORY: 18,000 Free Range birds in the same house, there was drop in egg production on Saturday, egg production had dropped from 90% to 60% in 48 hours. The feed intake was down 40g/head, mortality- within normal limits<br>Birds in one corner of the house seemed quieter but none sick in appearance. In this corner the egg-shell quality was poor.<br>See also PVir/20/00192<br>GROSS NECROPSY:<br>Active ovarian follicles. No or partially formed eggs in oviduct.<br>LABORATORY TEST RESULTS:<br>AI MP, and H6N1 PCR positive, H5 H7 PCR negative<br>Note that Brain samples have tested low positive and high positive in the rest of tissues.
14/03/2020	Grandparent	Northern Ireland	Co. Fermanagh	5,600	2020-004852	*H6N1*	Birds @ 56 weeks, 15 deaths, egg drop and a green scour. This was a grandparent site. It satisfies the case criteria.
16/03/2020	Commercial layers	Scotland	Scottish borders	20,000	DPR2020/09	*H6N1*	A private veterinary surgeon reported suspicion of Notifiable Avian Disease on 73 week old commercial layers (table eggs) on a free range unit in Scotland. This premises was part of the same group of farms linked to DPR 2020 02, DPR 2020 04 and DPR 2020 07. There was only one house of 20,000 layers on the premises. Birds showed signs of reduced egg production, increased mortality, reduced feed and water intake and loose droppings. APHA Vet investigated and confirmed findings as above. No evidence of wet droppings. Birds all appeared bright, alert and responsive with no other clinical signs indicating there to be a problem apart from a dramatic decrease in egg production to 30% of what is expected. Samples were taken on suspicion of Notifiable Avian Disease.

							M gene (Influenza A) PCR POSITIVE; H5 PCR NEGATIVE, H7 PCR NEGATIVE L gene (Newcastle Disease) NEGATIVE. Serology Titres to Newcastle disease vaccination as expected. Haemagglutinating virus has been isolated after 2 days incubation. It has been identified as a virus of low pathogenicity, H6N1.
16/03/2020	Commercia l layers	Scotland	Scottish borders	32,000	DPR2020/10	*H6N1*	A private veterinary surgeon reported suspicion of Notifiable Avian Disease on 76 week old commercial layers (table eggs) on a free range unit in Scotland. This premises is part of the same group of farms linked to DPR 2020 02, DPR 2020 04, DPR 2020 07 and DPR 2020 09. There is only one house of 32,000 layers on the premises. Birds are showing signs of reduced egg production, increased mortality. APHA Vet has investigated and confirmed findings as above. Birds all appear bright, alert and responsive with no respiratory or nervous clinical signs. The droppings are slightly looser than normal, water consumption has decreased and mortality has doubled over the weekend to 60 dead overnight. Notifiable Avian Disease could not be ruled out and samples were taken.
							M gene (Influenza A) PCR POSITIVE; H5 PCR NEGATIVE, H7 PCR NEGATIVE, L gene (Newcastle Disease) NEGATIVE. Serology Titres to Newcastle disease vaccination, as expected. Haemagglutinating virus has been isolated after 2 days incubation. It has been identified as a virus of low pathogenicity, H6N1
19/03/2020	FR laying hens	RoI	Co. Monaghan	8,332	Dubl/20/00171	*H6N1*	HISTORY: 8332 birds on site, production etc normal until Sunday, some white eggs observed, feed intake decreased from 124g to 110g in 3 days, egg production decreased: Sun 7843, Mon 6806, Tues 4521, Wed very little eggs, increasing white, misshape, shelless, very soft-shelled eggs. LABORATORY RESULTS: samples have tested positive for H6N1 LPAI. Negative for H5 and H7.
23/03/2020	Layers	Northern Ireland	Co. Armagh	15,000	2020-005233	*H6N1*	A private veterinary practitioner reported a commercial egg laying flock, 2 house barn system unit. No mortality, birds looking normal etc. but egg drop with pale and jelly eggs. No diarrhoea.
03/04/2020	FR laying birds	RoI	Co. Monaghan	5,367	Dubl/20/00201	*H6N1*	HISTORY: 20 X Laying hens, 53 weeks old, clinically look ok, decreased egg production, a lot of white and soft-shelled eggs, suspect LPAI.<br>LABORATORY RESULTS: Samples have tested positive for AI MP and H6N1 PCR. Samples are negative for H5 and H7 PCR.
15/04/2020	Organic layers	RoI	Co. Monaghan	5,856	Dubl/20/00207	*H6N1*	HISTORY:<br>20 x layers, 39 weeks old, euthanised by cervical dislocation, decreased feed & water intake, increase in mortality, 33 in last week, none prior to this, decrease in egg production, from 55% down to 33% in 1 day, increase in white eggs, treatment given - neomay to treat suspect ecoli infection, finished on 8/4/20, suspect LPAI<br>GROSS NECROPSY:<br>Egg peritonitis identified in one, poor BCS and prominent keel bone.<br>Urates identified in one.<br>No other significant gross findings identified<br>LABORATORY TEST RESULTS: Samples have tested positive for AI MP PCR and H6N1 PCR. Brain is negative for AI PCR. Negative for H5 and H7 PCR.<br>DIAGNOSIS & COMMENT: LP H6N1

17/04/2020	Free Range Layers	RoI	Co. Monaghan	13,896	Dubl/20/00209	*H6N1*	HISTORY:20 x table egg layers, egg drop, white eggs, thin shells, min feed decrease, min water decrease, LPAI suspect<br>GROSS NECROPSY:<br>BCS moderate, fair preservation<br>Focally extensive moderate peritonitis identified in two.<br>No other significant gross lesions noted.<br>LABORATORY TEST RESULTS: Samples have tested positive for AI MP PCR, H6N1 PCR and negative for H5 and H7 PCR.<br>Brain is negative for Ai PCR.<br>DIAGNOSIS & COMMENT: LP AI H6N1
20/04/2020	14-week fattening turkeys	RoI	Co. Monaghan	6,010	Dubl/20/00213	*H6N1*	HISTORY: 19 x turkeys, slightly depressed, given Solamocta<br>GROSS NECROPSY: No significant gross lesions.<br>LABORATORY TEST RESULTS: 1 sample has tested positive for AI MP PCR and H6N1 PCR. Negative for H5 and, H7 PCR. The reminding samples have tested negative for AI PCR however positive for AI ELISA.<br>DIAGNOSIS & COMMENT: LP H6N1
24/04/2020	Free Range Table egg layers	RoI	Co. Monaghan	5,635	Dubl/20/00225	*H6N1*	HISTORY: 5,600 free range laying hens on site. Screened by private lab for AI due to proximity to other cases- pooled samples were PCR positive for AI. White eggs yesterday (5 dozen), increased to 20 dozen today, production decreasing, LPAI suspect?<br>LABORATORY TEST RESULTS: samples are positive for AI MP, H6N1 PCR. Brain is negative for Ai PCR.<br>Samples are negative for H5 and H7 PCR.<br>DIAGNOSIS & COMMENT: LP H6N1
06/05/2020	Organic laying hens	RoI	Co. Monaghan	6,000	Dubl/20/00234	*H6N1*	HISTORY: 20 x organic layers, 2 X 3000 houses organic laying hens, Screened negative for AI on samples taken last Tues 28th April in private lab.<br>54 weeks old, house 1, egg production down 45% over 2 days, white eggs and soft shelled eggs over last 2 days, mortality up to 8 per day, green diarrhoea, site in close proximity to LPAI Positive Site<br>Site is restricted and gave PH advice yesterday<br>GROSS NECROPSY:<br>All oviducts empty, active follicles, suppurative peritonitis in a few hens.<br>LABORATORY TEST RESULTS: Samples have tested positive for AI MP PCR, H6 N1 PCR. Low positive in Brain, high positive in the rest of tissues. Samples are negative for H5 and H7 PCR.<br>DIAGNOSIS & COMMENT: LP AI H6N1
15/05/2020	Laying Hens	RoI	Co. Monaghan	13,750	Dubl/20/00251	*H6N1*	HISTORY: 5 X Table Egg Layers, 67 weeks old, linked to previous AI Positive, same family.<br>GROSS NECROPSY:<br>Preservation and BCS moderate x 5<br>No significant gross lesions identified.<br>LABORATORY TEST RESULTS: some samples have tested AI H6N1 PCR positive. Negative in brain. Samples are negative for H5 and H7 PCR<br>DIAGNOSIS & COMMENT: LP H6N1

02/06/2020	Table Egg Layer	RoI	Co. Monaghan	N/A	Dubl/20/00273	*H6N1*	HISTORY:<br>Table egg laying hens, 54 weeks old, increase in mortality and white eggs, production down during last 3 days, private AI sampling positive, 49 losses in 3 days.<br>GROSS NECROPSY:<br>Preservation and BCS fair in birds 001 to 005<br>Focally extensive peritonitis in bird 003 c.2-3cm.<br>No further significant gross lesions identified<br>LABORATORY TEST RESULTS:<br>Samples have tested positive for AI MP, H6N1 PCR. Negative in Brain. Samples have tested negative for H5, H7 PCRs.<br>E.coli isolated from peritoneal swab<br>DIAGNOSIS: LP AI H6N1
19/06/2020	Fattening turkeys	RoI	Co. Monaghan	6,000	Dubl/20/00306	*H6N1*	HISTORY: 20 x 33-week-old turkeys, 6,000 birds on site in 2 houses, 1 affected.<br>GROSS NECROPSY: Wet cloacal in several submitted bird and marked pulmonary congestion and oedema in visceral sampled birds.<br>LABORATORY TEST RESULTS: Samples have tested positive for AI MP PCR, H6N1 PCR. Negative in Brain. Samples are negative for H5, H7 PCRs.<br>DIAGNOSIS & COMMENT: LPAI H6N1
