# Supplementary figures and images for: Evaluating the Impact of Low-Pathogenicity Avian Influenza H6N1 Outbreaks in United Kingdom and Republic of Ireland Poultry Farms during 2020
Source: Viruses. 2024 Jul 16;16(7):1147. doi: 10.3390/v16071147 (PMC11281592; doi:10.3390/v16071147)

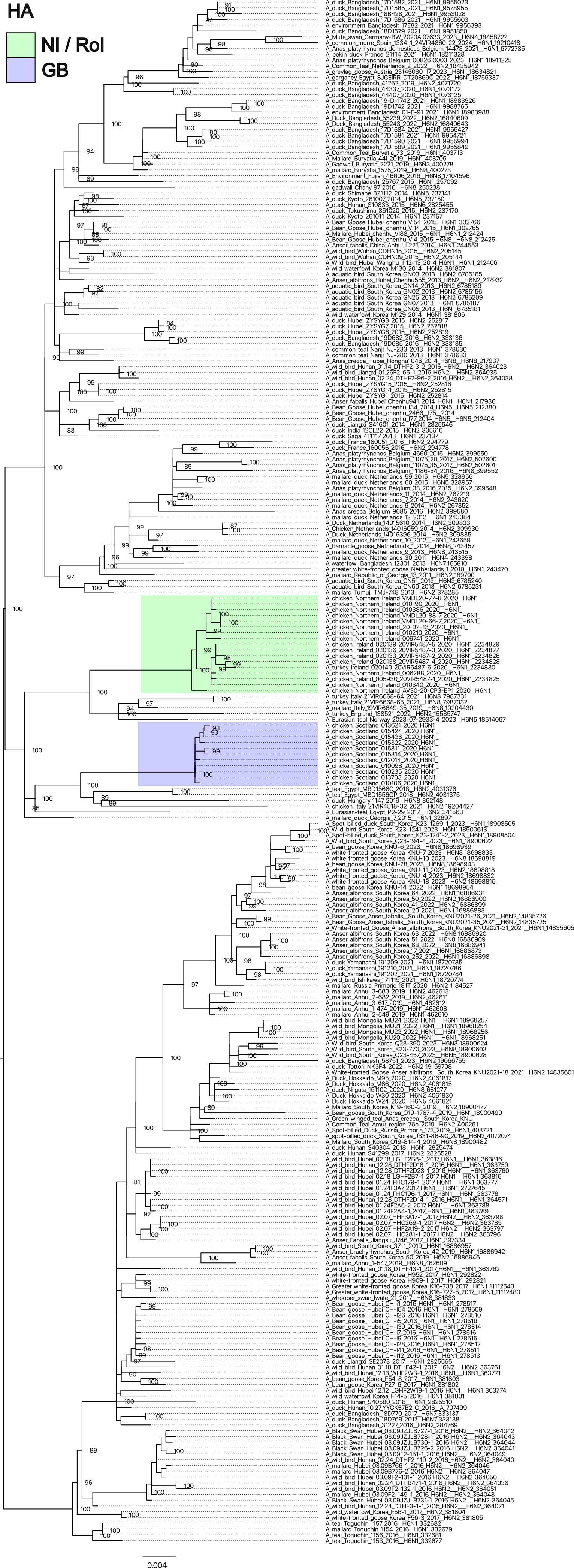

Supplement: Supplementary file 1 [file viruses-16-01147-s001.zip › Figure S1.png]

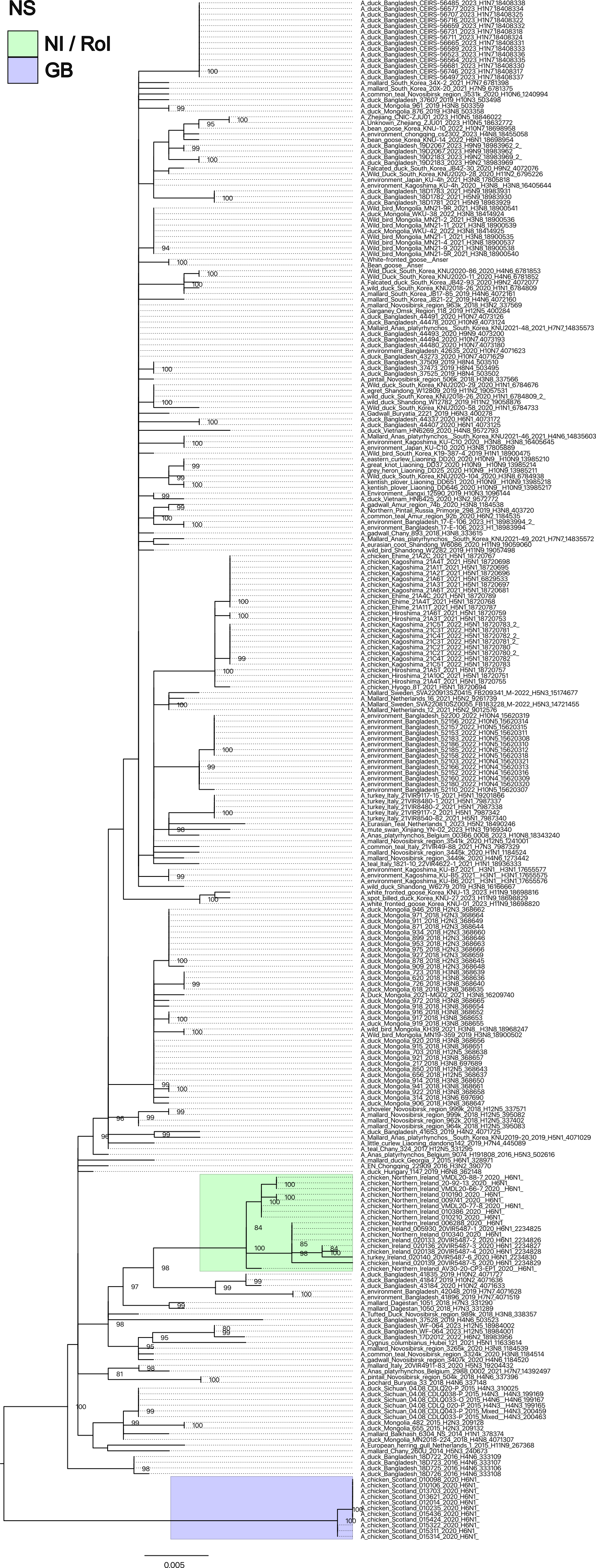

Supplement: Supplementary file 1 [file viruses-16-01147-s001.zip › Figure S8.png]

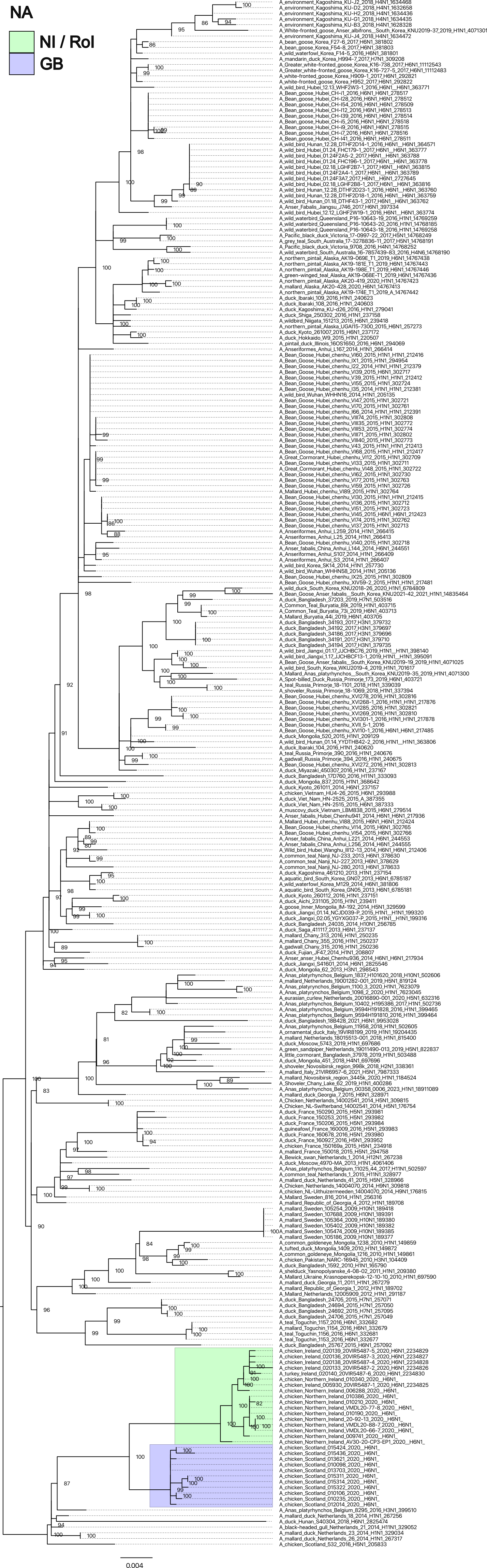

Supplement: Supplementary file 1 [file viruses-16-01147-s001.zip › Figure S2.png]

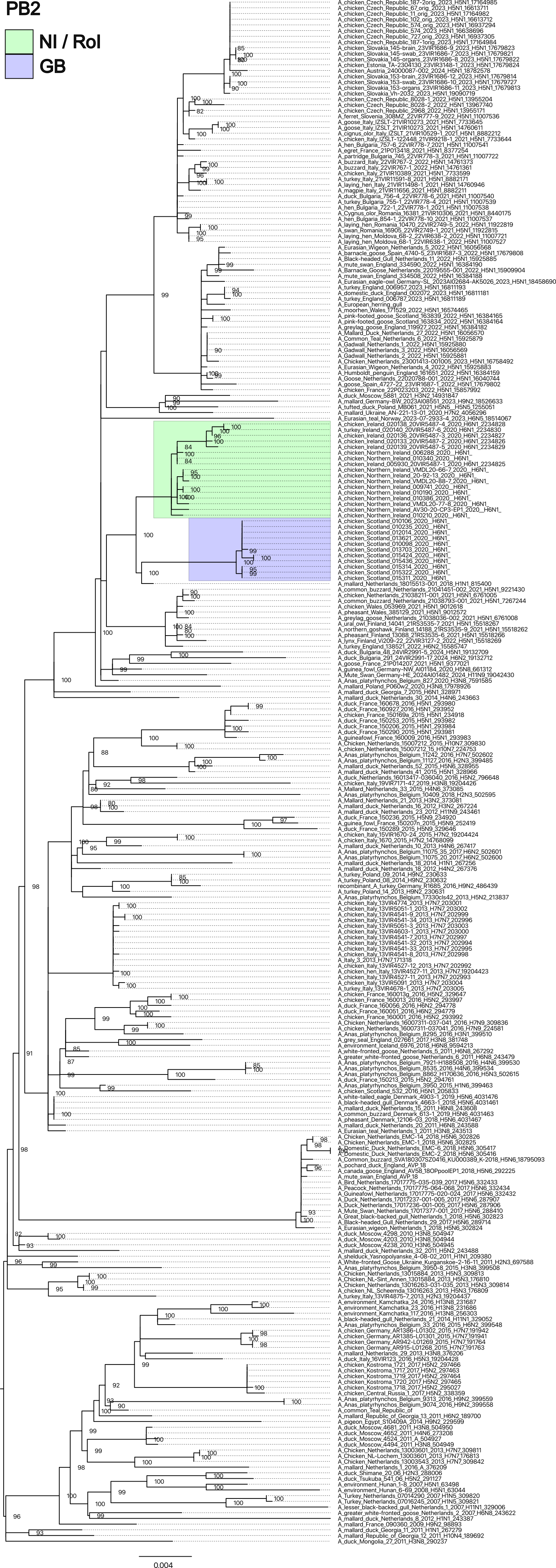

Supplement: Supplementary file 1 [file viruses-16-01147-s001.zip › Figure S3.png]

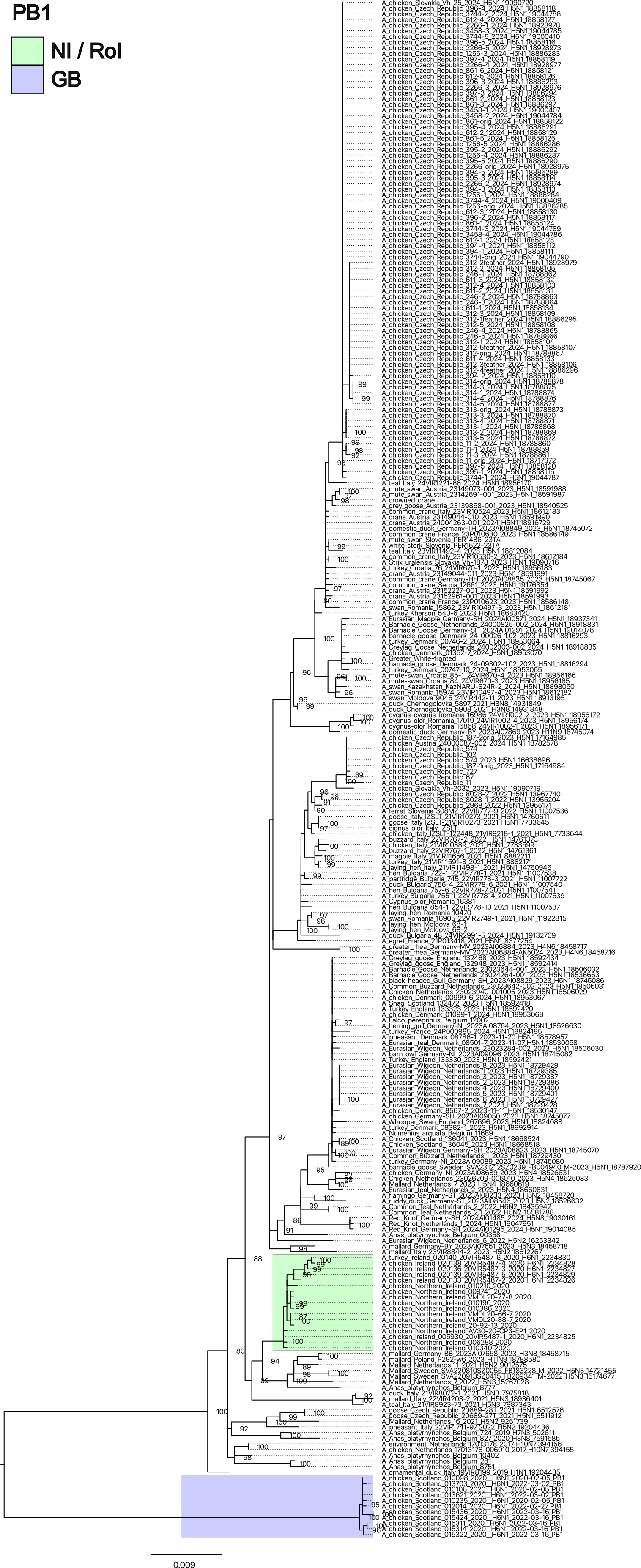

Supplement: Supplementary file 1 [file viruses-16-01147-s001.zip › Figure S4.png]

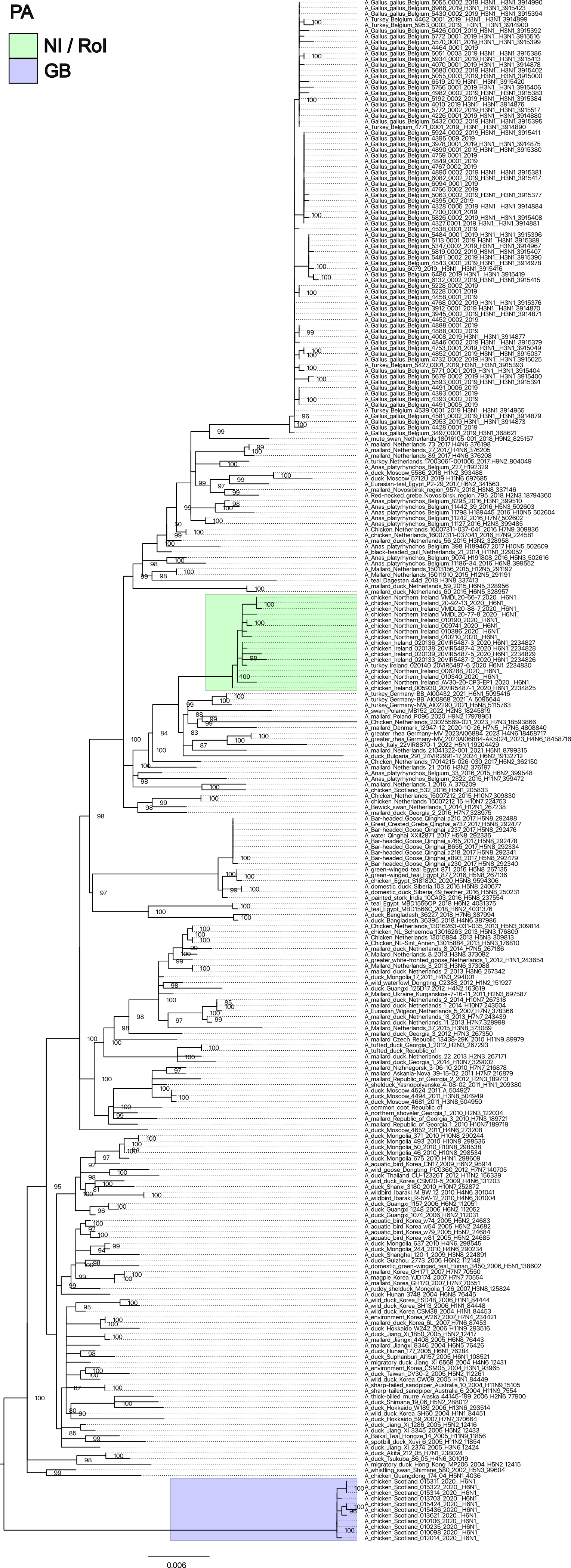

Supplement: Supplementary file 1 [file viruses-16-01147-s001.zip › Figure S5.png]

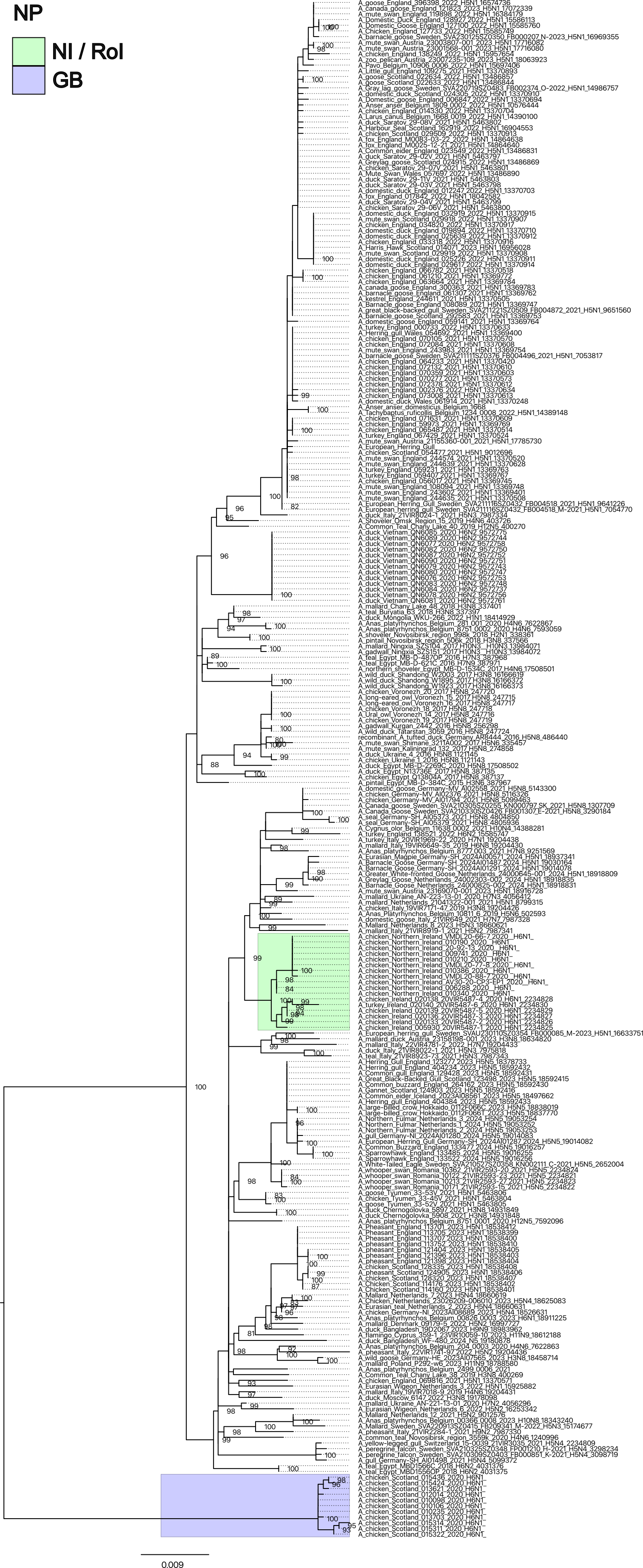

Supplement: Supplementary file 1 [file viruses-16-01147-s001.zip › Figure S6.png]

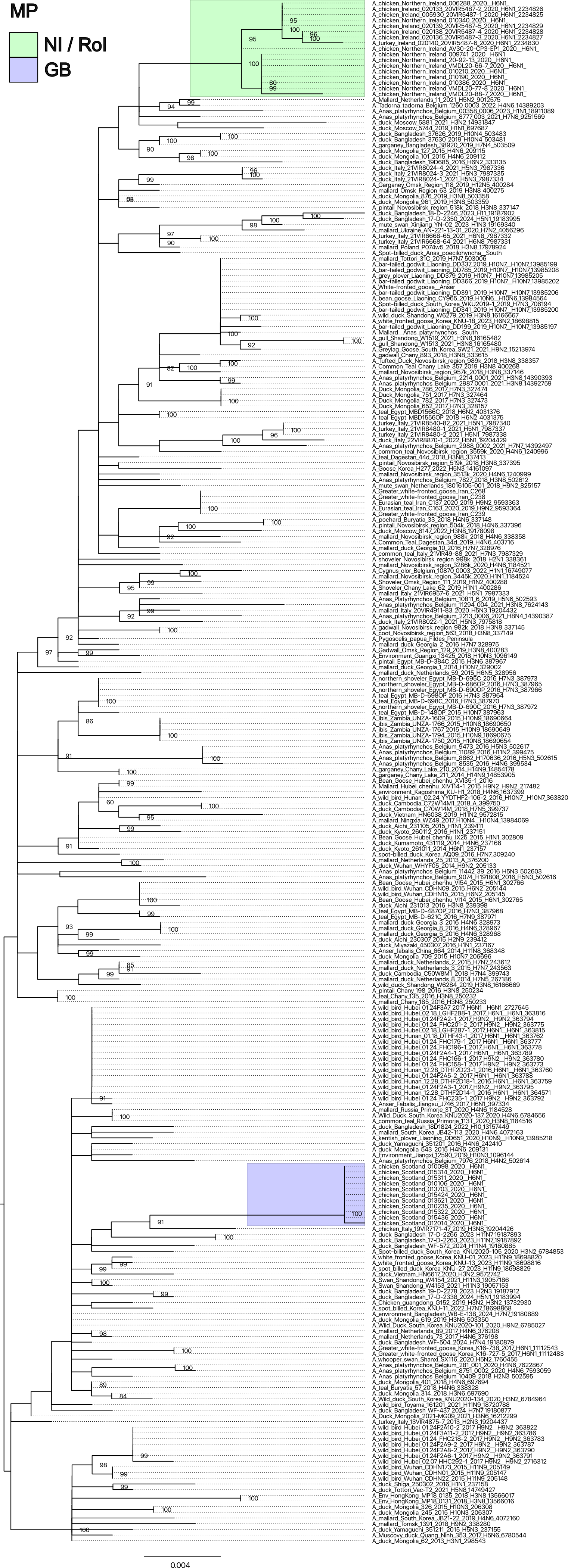

Supplement: Supplementary file 1 [file viruses-16-01147-s001.zip › Figure S7.png]
